# Focused ultrasound-induced blood-brain barrier opening promotes glioprotective phenotypes in ACSA-II+ murine astrocytes

**DOI:** 10.1016/j.isci.2025.113173

**Published:** 2025-07-22

**Authors:** Rebecca L. Noel, Alina R. Kline-Schoder, Alec J. Batts, Nancy Kwon, Fotios Tsitsos, Elisa E. Konofagou

**Affiliations:** 1 Department of Biomedical Engineering, Columbia University, New York, NY, USA; 2 Department of Radiology, Columbia University, New York, NY, USA

**Keywords:** Molecular biology, Neuroscience, Omics

## Abstract

Focused ultrasound-induced blood-brain barrier opening (FUS-BBBO) is a well-established neuroimmune modulation and drug delivery enhancement tool capable of effecting cellular, cognitive, and pathological benefits in the brain. However, the cellular mechanisms underlying these bioeffects remain incompletely characterized, motivating the present study investigating the role of a critical glial cell type: astrocytes. Here, we use single-cell RNA sequencing and flow cytometry to characterize the phenotypic response of hippocampal astrocytes 2, 4, and 7 days after FUS-BBBO exposure in aged WT and Alzheimer’s disease mice. The data presented herein indicate that FUS-BBBO increases gene expression related to synapse modification, neurogenesis, ion transport, and neuronal development. Taken together, these data elucidate a glioprotective role for hippocampal astrocytes following FUS-BBBO, offering mechanistic context and support for previously observed benefits of FUS-BBBO. This evidence critically deepens our understanding of the cellular effects of FUS-BBBO as clinical adoption becomes more widespread.

## Highlights

• FUS-BBBO promotes hippocampal astrocytes glioprotection without proliferation
• FUS-BBBO induces acute synapse modification and sustained cell component movement
• FUS-BBBO increases astrocytic signaling for neurogenesis and OPC differentiation
• FUS-BBBO boosts supportive sub-population prevalence while decreasing neurotoxic

## Graphical abstract

**Figure:**
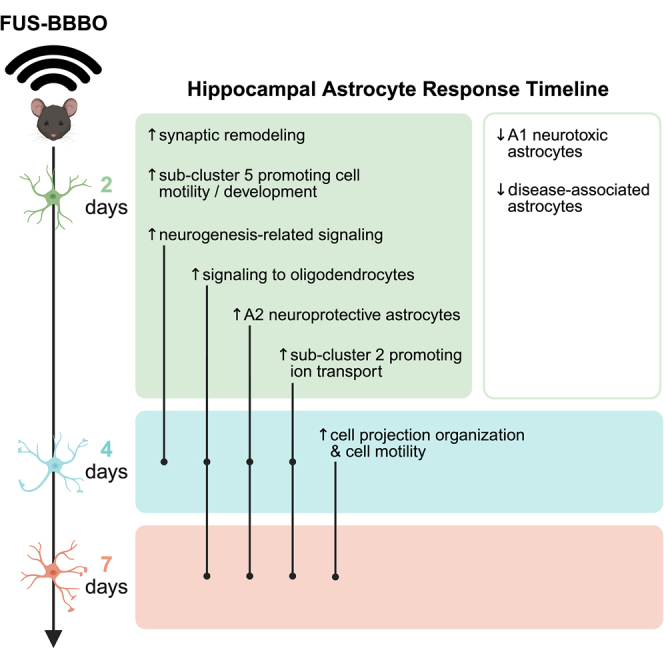

## Introduction

### Astrocytes

Astrocytes are glial cells of the central nervous system (CNS) that provide structural, trophic, and homeostatic support to adjacent cells. Astrocytes account for the largest fraction of cells in the brain and are heterogeneous in their morphological, transcriptomic, and functional phenotypes.^1^^,^^2^^,^^3^ They comprise an essential component of the neurovascular unit (NVU), facilitating signal transduction, regulating synaptic neurotransmitter concentration, signaling between the CNS and cerebral vasculature, and regulating synaptic plasticity.^1^^,^^4^ Astrocytes participate actively in neuronal signaling as the third component of tripartite synapses with pre- and post-synaptic neurons. Their proximity to neuronal synapses not only enables astrocytes to sense and modulate synaptic activity but also to direct synapse formation, elimination, and plasticity.^5^ This synaptic maintenance may be facilitated by either gliotransmitter or contact-mediated signaling via perisynaptic astrocytic processes (PAPs).^1^^,^^6^ PAPs physically associate with neuronal synapses and house functional membrane proteins, such as glutamate transporters, to facilitate neurotransmitter uptake and recycling.^6^ Notably, synaptic dysfunction, and specifically changes in synaptic plasticity, has been linked to several neurodegenerative and cognitive disorders, especially Alzheimer’s disease (AD).^7^^,^^8^ Thus, astrocytic maintenance of synaptic plasticity may confer neuroprotective benefits, particularly in natural aging and AD. In addition to their association with neuronal synapses, astrocytes are physically incorporated into the blood-brain barrier (BBB), serving as a liaison for communication between neurons and cerebral vasculature. In particular, astrocytes have been identified as critical mediators of neurovascular coupling, wherein neurons signal for changes in cerebral blood vessel diameter.^9^^,^^10^ Conversely, in response to changes in cerebral vasculature astrocytes may affect neuronal signaling, specifically via glutamate, thereby facilitating bidirectional communication between neurons and the BBB.^11^

Historically, astrocytic activation has been categorized into one of two activation states: neurotoxic A1 or neuroprotective A2.^3^ Although this binary classification system fails to reflect the complexity and heterogeneity of astrocytic activation, it offers a useful framework for interpreting transcriptomic changes observed in activated astrocytes. A1 astrocytes are considered maladaptive as they may confer toxicity to neurons and synapses via neurotoxin secretion and loss of homeostatic functioning.^3^ Conversely, A2 astrocytes may improve neuronal health and survival by upregulating anti-inflammatory genes and producing neurotrophic factors.^3^ Although the specific gene signatures of A1 or A2 astrocytes vary depending on the activation stimulus, some of the most common markers used to identify these states are *C3*, *Serping1*, and *Psmb8* for A1 astrocytes, and *S100a10*, and *Stat3* for A2 astrocytes.^3^^,^^12^ A distinct population of disease-associated astrocytes (DAA) resulting from Alzheimer’s pathology in a mouse model of AD has been identified and transcriptomically characterized by Habib et al.^13^ This population, which appears early in AD mouse models and increases in abundance with age, is defined by high expression of the following markers: *Ggta1*, *Gsn*, *Osmr*, *Vim*, *Serpina3n*, *Ctsb*, and *Gfap*.^13^ Interestingly, this population of astrocytes has also been identified in aged, nonpathological mouse and human brain tissue, indicating that both age and AD may give rise to similar reactive astrocyte phenotypes.^13^

Astrocytes may also communicate with other CNS cells, including neurons, other astrocytes, oligodendrocytes, endothelial cells, and microglia via multiple mechanisms. Astrocyhtic signaling via secreted factors is one such mechanism, by which astrocytes may chemically mediate the functioning of the BBB by triggering intracellular changes or recruiting support cells to address disturbances.^14^^,^^15^ A role for astrocytes in mediating adult neurogenesis has been identified in both healthy and AD conditions.^16^ Astrocytic signaling may also initiate the differentiation and proliferation of oligodendrocyte precursor cells (OPCs) into mature, myelinating oligodendrocytes.^17^

The phenotypic and functional heterogeneity of astrocytic sub-populations necessitates the use of high-resolution, single-cell RNA sequencing to detect and discern the response of these cells to FUS-induced blood-brain barrier opening (FUS-BBBO).

### Focused ultrasound-induced blood-brain barrier opening

Focused ultrasound (FUS) involves the focusing of acoustic pressure waves in a 3D volume from an ultrasound transducer. When paired with systemically injected microbubbles, FUS can be used to open the BBB in a targeted and transient manner (FUS-BBBO). The safety of this technique has been demonstrated in many small animal models, nonhuman primates and is actively undergoing clinical evaluation.^18^^,^^19^^,^^20^^,^^21^^,^^22^^,^^23^^,^^24^ In addition to enhancing drug delivery efficiency across the BBB, FUS-BBBO alone has demonstrated profound protective and therapeutic benefits in murine models of AD.^23^^,^^25^^,^^26^^,^^27^ These studies demonstrate not only pathological improvement but also anxiety amelioration, spatial memory improvement, and enhanced reversal learning capacity.^23^^,^^25^^,^^27^ The mechanisms by which FUS-BBBO confers these favorable bioeffects is under active investigation. However, the discoveries that FUS-BBBO induces adult neurogenesis and neuroimmune activation offer considerable clarity toward a more complete understanding.^28^^,^^29^^,^^30^^,^^31^

### AD and astrocytes

AD is a neurodegenerative disease characterized by amyloid-β plaque and hyperphosphorylated tau accumulation.^32^ Clinically, AD patients present cognitive deficits, agitation and anxiety. On a cellular level, AD has been linked to a reduction in excitatory, glutamatergic synapses both in humans and in mouse models of AD.^33^ A loss of glutamate transporters VGLUT1 and VGLUT2 has been observed in AD, suggesting a link between the effective regulation of synaptic glutamate concentrations and the extent of neurodegenerative disease.^34^ AD progression is also associated with a reduction in synaptic plasticity and of synaptic density, which is associated with cognitive decline and may be a consequence of astrocyte atrophy.^35^^,^^36^ Astrocytes may play an active role in mitigating the symptoms and progression of AD via protective mechanisms but may also be responsible for disease progression due to loss of function and excessive inflammation.^12^^,^^36^ In terms of glioprotection against AD, astrocytic *Lrp1* plays a critical role in mediating amyloid uptake and metabolism.^37^ Synaptic regulation leading to enhanced plasticity and enhanced neuronal functioning may also confer protection against neurodegeneration and disease progression.

## Results

### FUS-BBBO induces dynamic responsiveness in hippocampal astrocytes

**Figure 1:**
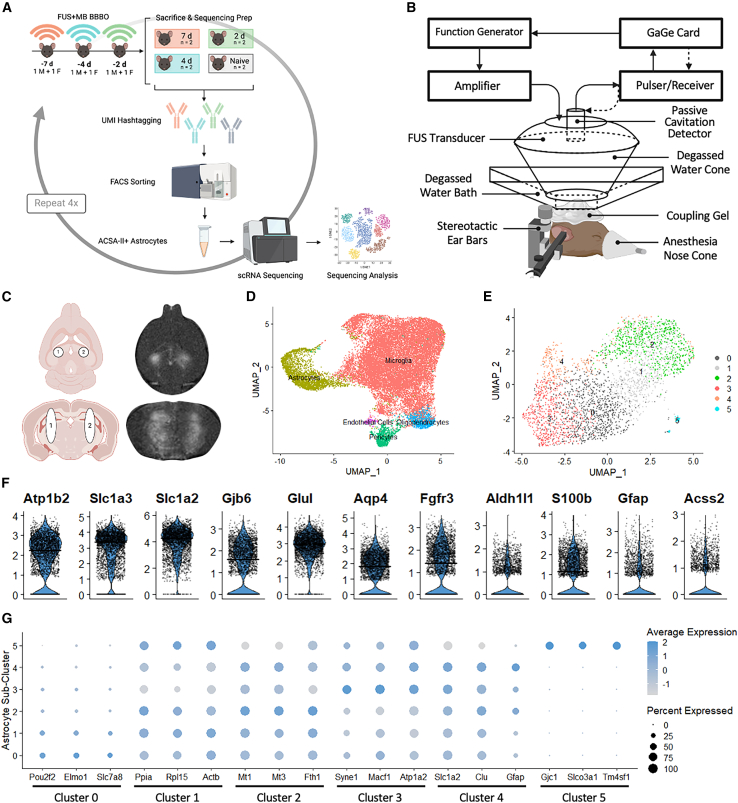
Overview of transcriptomic experiment cell populations (A) Single-cell RNA sequencing experimental overview. (B) Focused ultrasound setup. (C) Single, bilateral hippocampus FUS-BBBO targets schematic and T1-weighted MR image. (D) UMAP displaying cell-type clustering with labels based on canonical markers. (E) Astrocytic sub-clusters identified by cluster labels 0–5. (F) Expression of canonical astrocytic makers across astrocyte population. (G) Dot plot displaying prominence and extent of expression of most significant upregulated genes differentiating each astrocytic sub-cluster.

**Figure 2:**
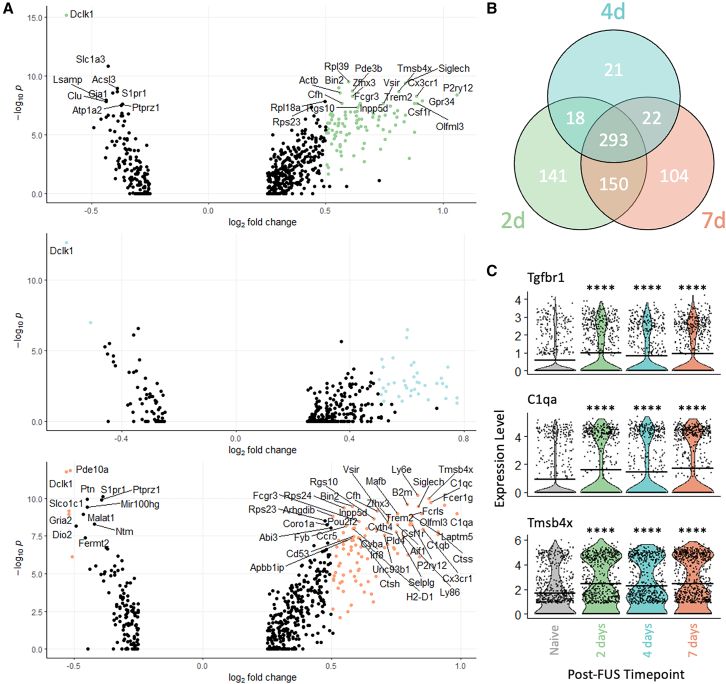
FUS-BBBO induces dynamic astrocytic response at short- and long-term time points (A) Volcano plots showing differential gene expression at 2, 4, and 7 days post-FUS-BBBO compared to naive controls. Colored dots represent differentially expressed genes with an adjusted *p* value < 0.05 and a log2(fold change) > 0.05. Labeled genes are differentially expressed with an adjusted *p* value < 5 × 10^−8^. (B) The number of significant differentially expressed genes unique to each time point and shared between different time points post-FUS-BBBO. (C) Violin plots showing representative differentially expressed genes at all three time points post-FUS-BBBO. Significant differences at each time point compared to naive are measured by Wilcoxon rank-sum test. ∗∗∗∗*p* ≤ 0.0001.

The application of FUS-BBBO in the hippocampus of 10-week-old WT mice induces dynamic, transcriptomic responsiveness as observed with single-cell RNA sequencing. Hippocampal astrocytes, which were sorted based on their expression of ACSA-II via fluorescence-activated cell sorting (FACS) (Figure 1A), were identified based on the expression of canonical astrocytic markers *Aqp4*, *Aldh1l1*, *S100b*, *Gfap* and others (Figure 1F). When compared to untreated, age-matched control groups, the astrocytes measured 2, 4, and 7 days post-FUS-BBBO all exhibited significant differentially expressed genes (Figure 2A). The large number (293) of differentially expressed genes shared between all three time points indicates that an early and sustained astrocytic response results from exposure to FUS-BBBO (Figure 2B). *Tgfbr1*, *C1qa*, and *Tmsb4x* represent interesting genes related to astrocyte reactivity that are significantly upregulated at all three time points post-FUS-BBBO (Figure 2C).

### FUS-BBBO induces astrocyte reactivity without significant gliosis

**Figure 3:**
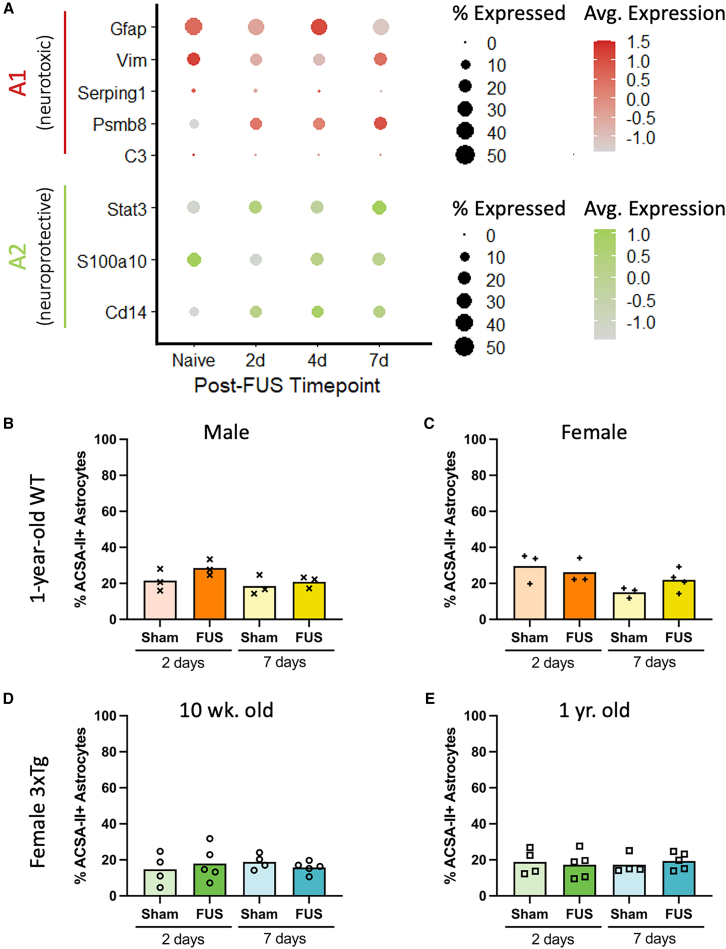
FUS-BBBO induces astrocytic activation without significant proliferation (A) Dot plots showing the percentage of astrocytes expressing and relative expression levels of each gene associated with classic A1 (neurotoxic) and A2 (neuroprotective) astrocytic activation states. (B–E) The percentage of ACSA-II+ astrocytes out of all live cells is shown for 1-year-old WT male (B), 1-year-old WT female (C), 10-week-old 3xTg female (D), and 1-year-old female mice (E). Comparisons between time point-matched sham and FUS groups are not significant (*p* > 0.05) by unpaired t test (B–E).

Astrocytic populations are extremely heterogeneous, with activation states that may have protective, deleterious, or mixed effects on the surrounding tissue. Although a binary categorization system is insufficient for capturing the complexity of astrocyte populations *in vivo*, classifying astrocytes as A1 (neurotoxic) or A2 (neuroprotective) is useful for broadly understanding the transcriptomic and morphological changes that astrocytes undergo in response to a stimulus.^3^ Evaluation of the changes in gene expression across all hippocampal astrocytes isolated from treated animals reveals a mixed transcriptomic response profile to FUS-BBBO. The changes in classic A1-associated genes *Gfap*, *Vim*, *C3*, *Psmb8*, and *Serping1* are shown in Figure 3. Interestingly, of the five genes presented here, three of them, *Vim*, *Serping1*, and *C3* are downregulated relative to control astrocytes after exposure to FUS-BBBO. Additionally, expression of *Gfap*, perhaps the most popular marker of astrocyte reactivity, peaks at 4 days post-FUS and returns to a low level at 7 days post-FUS-BBBO. Classic A2-associated genes *Stat3*, *S100a10*, and *Cd14* are also presented in Figure 3. Of these three genes associated with neuroprotective activation, relative expression of two, *Stat3* and *Cd14*, increases after FUS-BBBO exposure. Taken together, the downregulation of A1, and upregulation of A2-associated astrocytic markers indicates that overall, the population-wide response in hippocampal astrocytes tends toward a glioprotective phenotype.

Interestingly, flow cytometry analysis of ACSA-II+ astrocytes reveals no significant increase in the percent of astrocytes out of all live cells at short- (2 days) and long-term (7 days) time points after FUS-BBBO across aged WT male and female mice, as well as young and aged 3xTg-AD female mice (Figures 3B–3E). In aged, 1-year-old WT animals there were no significant differences detected between anesthesia sham animals and FUS-BBBO-treated animals at 2 or 7 days after FUS-BBBO by one-way ANOVA with multiple comparisons (Figures 3B and 3C). This lack of significant difference was observed both in WT male (Figure 3B) and female (Figure 3C) animals, indicating no sex-dependent difference in this response. Further, a lack of significant change in population percentage was also observed in both young, 10-week-old (Figure 3D) and aged, 1-year-old (Figure 3E) 3xTg-AD female mice. Taken together, the observed increase in astrocytic activation markers without an observed increase in population percentage even with natural aging and AD pathology offers promising support for a FUS-induced increase in beneficial astrocyte activation without triggering significant astrocyte proliferation implicated in glial scarring.

### Gene ontology analysis of FUS-BBBO treated astrocytes indicates early upregulation of synaptic regulation

**Figure 4:**
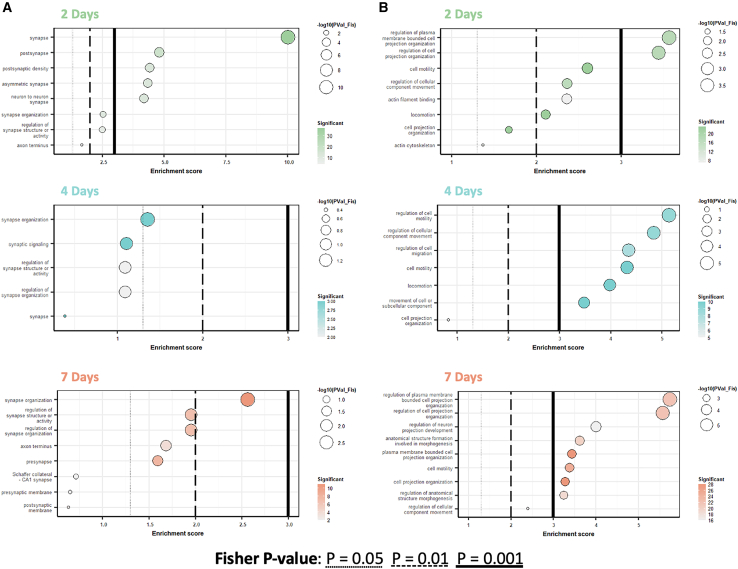
Gene ontology analysis reveals early increase in astrocytic synapse modification and sustained cellular component movement after FUS-BBBO (A and B) Gene ontology terms related to synapse modification (A) and cellular component movement (B) to which differentially expressed genes were annotated for astrocytes 2, 4, and 7 days post-FUS-BBBO. Bubble size indicates the -log_10_(Fisher *p* value) and color scale indicates the number of significant genes annotated to each term. Solid, dashed, and dotted lines indicate Fisher *p* values of 0.001, 0.01, and 0.05, respectively.

Gene ontology (GO) analysis of FUS-BBBO-treated astrocytes gives rise to a prominent trend in GO terms related to synapse modification and maintenance. Several terms such as “synaptic organization,” “regulation of synaptic structure or activity,” and “pre-” and “post-synaptic membrane” are significantly upregulated at 2, 4, and 7 days post-FUS-BBBO (Figure 4A). Synapse-related terms are most significantly upregulated at the acute, 2-day post-FUS time point; however, all three time points exhibit transcriptional changes annotated to synaptic maintenance. This finding indicates that the early astrocytic response to FUS-BBBO is dominated by synaptic maintenance, which may include regulation of synaptic proteins that affect the probability of neurotransmitter transmission, the engulfment of synapses in synaptic pruning, or the production of factors that promote synaptogenesis. The activation of hippocampal astrocytes by FUS-BBBO overwhelmingly affects the synapses on which these astrocytes act, offering the opportunity to make these synapses more efficient and optimize hippocampal neural circuits.

### GO analysis of FUS-BBBO treated astrocytes indicates delayed and sustained upregulation of cytoskeletal movement and reorganization

In addition to GO terms related to synapse regulation and modification, the changes in gene expression relative to untreated controls also demonstrated prominent annotation to terms related to cellular component movement (Figure 4B). In contrast to the terms related to synapse modification, terms related to cytoskeletal movement are more significantly upregulated at 4 and 7 days than at 2 days, suggesting that this process may follow later in the response timeline to FUS-BBBO, and may be sustained for a longer period post-treatment. Terms such as “cell projection organization,” “cell motility,” and “movement of cell or subcellular component” are significantly upregulated relative to the untreated control animals, indicating that affected astrocytes may undergo process extension or movement in response to FUS-BBBO. This cytoskeletal movement may be implicated in many different processes, including modulation of synapse monitoring and contact, process extension, and more, which contribute to homeostatic maintenance.

### FUS-BBBO induces upregulation of genes related to neurogenesis

**Figure 5:**
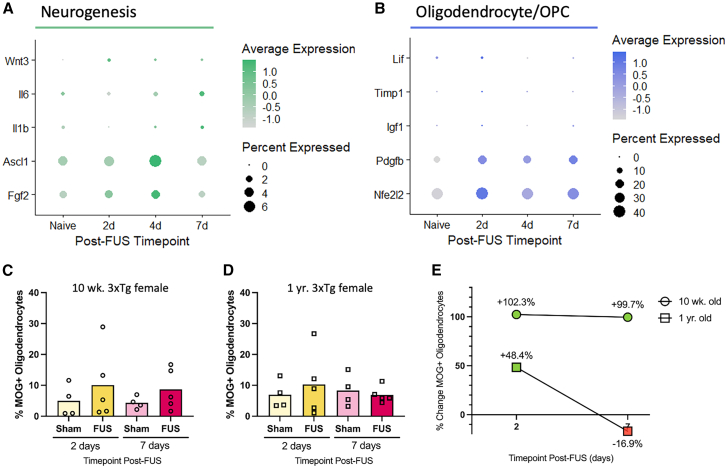
Astrocytes may orchestrate diverse glioprotective processes involving neurons and oligodendrocytes after FUS-BBBO exposure (A) Dot plot showing the expression of genes implicated in astrocytic direction of neurogenesis in naive and FUS-treated astrocytes. (B) Dot plot showing the expression of genes related to astrocyte signaling to OPCs and oligodendrocytes in naive and FUS-treated astrocytes. Dot size corresponds to the percentage of astrocytes expressing a given gene and the color bar indicates to the relative expression level of each gene in (A) and (B). (C and D) The percent of MOG+ oligodendrocytes out of all live cells isolated from 10-week-old (C) and 1-year-old (D) 3xTg mice. Time point-matched comparisons between anesthesia sham and FUS-treated groups are not significant (*p* > 0.05) by unpaired t test. (E) The percent change in FUS-BBBO groups compared to time point-matched anesthesia groups in the percentage of MOG+ oligodendrocytes at 2 and 7 days post-intervention.

The application of FUS-BBBO *in vivo* gives rise to upregulated factors related to and required for neurogenesis. Namely, the expression of *Wnt3*, *Il6*, *Il1b*, *Fgf2*, *Ascl1*, and *Sirt2* were upregulated in astrocytes isolated from FUS-BBBO treated animals at 2, 4, and 7 days post-FUS-BBBO (Figure 5A). Although expressed in only a small percentage of the entire population of hippocampal astrocytes, the upregulation of *Wnt3* expression relative to untreated controls indicates a positive trend in favor of increased, astrocyte-mediated neurogenesis in the hippocampus. Although also expressed by only a small fraction of the total astrocyte population, *Fgf2* expression is increased, not only in the expression level but also in the fraction of astrocytes expressing it. *Fgf2* expression increases at 2 and peaks at 4 days post-FUS, returning to baseline, naive levels 7 days after FUS-BBBO. This trend indicates a potential transient response and upregulation in the promotion of new neuronal stem cells after FUS-BBBO. Finally, the upregulation *Ascl1* expression, peaking at 4 days post-FUS indicates a positive trend promoting neurogenesis in affected astrocytes after FUS-BBBO (Figure 5A).

### FUS-BBBO promotes astrocytic signaling to promote oligodendrocyte support and differentiation

FUS-BBBO gives rise to an upregulation of signaling molecules linked to astrocytic communication with oligodendrocytes. FUS-BBBO induces an acute increase in *Lif* expression 2 days after FUS-BBBO exposure, indicating that hippocampal astrocytes may transiently boost local myelination by surrounding oligodendrocytes, favorably contributing to greater neuron support and insulation, particularly in neurodegenerative and demyelinating conditions. The data presented herein indicate that hippocampal astrocytes in FUS-treated mice undergo a transient increase in *Timp1* expression relative to untreated controls (Figure 5B). This increase is observed at 2 days post-FUS-BBBO, but expression levels return to baseline by 4 and 7 days post-FUS-BBBO. An additional astrocytic factor, insulin-like growth factor-1 (*Igf1*), known to boost oligodendrocyte populations and myelination, is expressed by only a small fraction of the total astrocyte population in the present study; however, the acute upregulation of *Igf1* 2 days post-FUS-BBBO indicates a positive trend in astrocyte-orchestrated myelination. In demyelinating conditions astrocytic secretion of growth factors may promote OPC maturation and subsequent myelination. In response to FUS-BBBO specifically, the expression of two such growth factors, platelet-derived growth factor beta (*Pdgfb*) and fibroblast growth factors (*Fgf2*), increases compared to naive animals (Figure 5B). The expression of *Fgf2* increases acutely, peaking at 4 days post-FUS-BBBO, while the increase of *Pdgfb* is sustained at 2, 4, and 7 days post-FUS-BBBO. This indicates that FUS-BBBO promotes an activated phenotype supportive of OPC differentiation in hippocampal astrocytes, which may contribute to remyelination, insulation or repair of existing neurons. This is especially relevant in the case of neurodegenerative disease where the myelin sheath has been damaged or degraded, leading to neurodegeneration and subsequent cognitive decline.

The percentage of MOG+ oligodendrocytes out of total live cells was quantified using flow cytometry in 10-week-old (Figure 5C) and 1-year-old (Figure 5D) female, 3xTg-AD mice. Quantification of these cells revealed no statistically significant differences between treatment groups, thus the observed differences between groups should be interpreted only as trends that may indicate a biological effect. This quantification revealed an increase in the percentage of MOG+ oligodendrocytes at both 2 days and 7 days post-FUS-BBBO in 10-week-old animals compared to time point-matched anesthesia sham controls. Differences between time point-matched anesthesia and FUS-treated animals were not significant by unpaired t test. The percentage of MOG+ oligodendrocytes in the 1-year-old 3xTgs increased 2 days post-FUS, but seemed to return to baseline anesthesia sham levels at 7 days. The differences between time point-matched anesthesia sham and FUS-BBBO groups were also not significant by unpaired t test for the 1-year-old groups. Interestingly however, calculating the percent change in the percent of MOG+ oligodendrocytes in FUS-treated compared to naive animals reveals an interesting trend in the relative effectiveness of astrocytic activation of OPCs in young compared to aged and progressed 3xTg-AD animals. Namely, a 102.3% increase in MOG+ oligodendrocytes was observed 2 days after FUS-BBBO in 10-week-old females, while only at 48.8% increase was observed in 1-year-old animals (Figure 5E). This increase in MOG+ oligodendrocytes was sustained at 7 days post-FUS-BBBO in 10-week-old animals, where a 99.7% increase was observed compared to 7-day anesthesia shams. However, 1-year-old animals reverted to baseline sham levels, with a 16.9% fewer MOG+ oligodendrocytes 7 days post-FUS-BBBO compared to the anesthesia sham group. Overall, FUS-BBBO induced a more extensive and sustained increase in MOG+ oligodendrocytes in young, 10-week-old 3xTg mice compared to aged and more pathologically progressed 1-year-old 3xTg mice. Taken together, this indicates that astrocytic signaling to OPCs may have a greater, sustained effect in young, early AD mice than in those who are aged with significant AD progression.

### Heterogeneous astrocyte population evolves toward increased signaling and glioprotective phenotypes following FUS-BBBO

**Figure 6:**
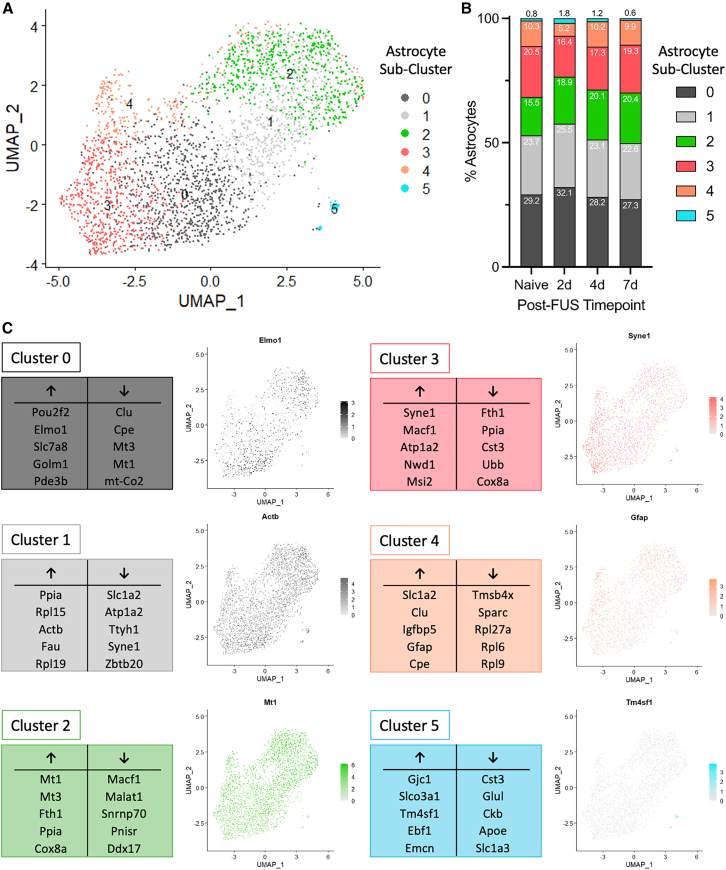
Astrocyte sub-clusters exhibit unique gene expression signatures (A) A UMAP illustrating six astrocyte sub-clusters based on differential gene expression profiles. (B) The percentage of each sub-cluster comprising astrocytes isolated from naive and FUS-treated animals 2, 4, and 7 days post-FUS-BBBO. (C) The most significant up- and down-regulated genes for each of the six astrocyte sub-clusters are shown, along with a feature plot illustrating relative expression of one of these genes across the astrocyte sub-cluster UMAP. See also Table S1.

Astrocytes are a highly heterogeneous cell type with specialized roles depending on their location in the brain and specialized function. Thus, evaluation of the sub-populations of hippocampal astrocytes and their dynamics over time following exposure to FUS-BBBO is warranted for a more complete understanding of the astrocytic response to FUS. Using nonlinear dimensional reduction clustering techniques, six distinct astrocyte sub-clusters emerged from the isolated astrocytes in each condition group (Naive, 2 days, 4 days, and 7 days post-FUS) (Figure 6A). The percentage of total astrocytes in each sub-cluster is shown in Figure 6B. Each of these sub-clusters has an associated gene signature, with up or down regulation of a unique signature set of genes (Figure 6C). The five most significantly up- and down-regulated genes differentiating each cluster are shown in Figure 6C, along with a feature plot illustrating relative expression of one of these genes on the uniform manifold approximation and projection (UMAP) visualization of all isolated astrocytes. Representative GO annotations are also shown in Table S1. Investigating individual genes that are up- and down-regulated in these clusters, in addition to performing GO analysis on their expression profiles, reveals a distinct function for each of these clusters within the hippocampus.

### FUS-BBBO decreases proportion of disease-associated astrocyte sub-population

**Figure 7:**
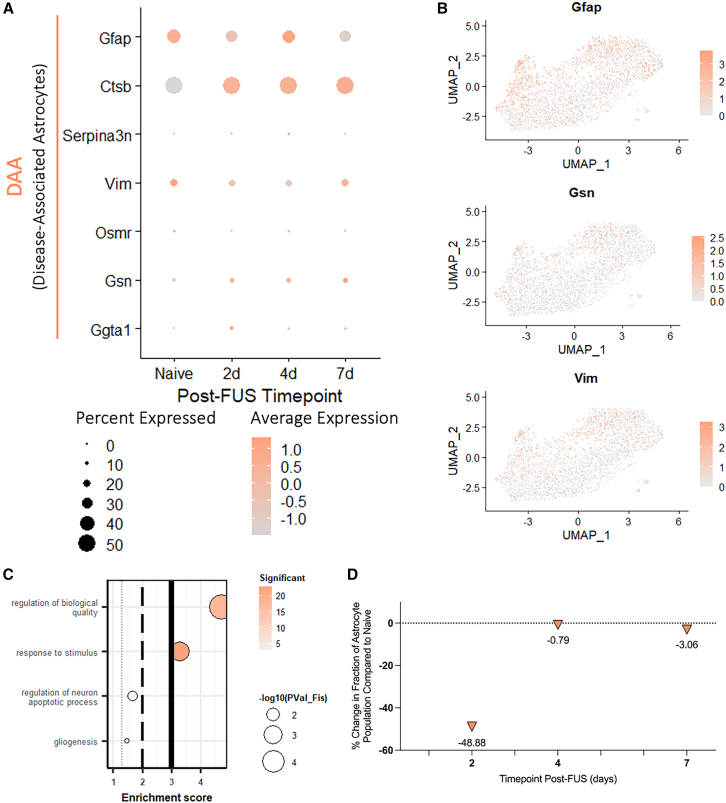
FUS-BBBO decrease the proportion of sub-cluster resembling disease-associated astrocyte phenotype (A) Dot plot illustrating relative expression of disease-associated astrocyte markers as identified by Habib et al., 2020. Dot size indicates the percent of astrocytes expressing each gene and dot color indicates relative expression. (B) Feature plots for three disease-associated astrocyte markers, *Gfap*, *Gsn*, and *Vim* are shown on the astrocyte sub-cluster UMAP, localizing primarily to sub-cluster 4. (C) Gene ontology terms indicative of stress response are annotated to and shown for the sub-cluster 4 gene expression profile. Dot size indicates the -log_10_(Fisher *p* value) and dot color indicates the number of significant genes annotated to each term. (D) The percent change in the fraction of astrocytes in sub-cluster 4 is shown at 2, 4, and 7 days post-FUS-BBBO compared to naive.

Disease-associated astrocytes (DAA) have been identified as a sub-population of reactive astrocytes found in aged WT and 3xTg-AD animals by Habib et al.^13^ The expression of these DAA genes, such as *Gfap*, *Serpina3n*, *Vim*, and *Osmr* are expressed by a relatively small number of the young, WT astrocytes included in the single-cell sequencing data analyzed here; however, interestingly, the expression of these genes is reduced over time after exposure to FUS-BBBO relative to naive controls (Figure 7A). Further, the expression of these markers appears to be concentrated in sub-cluster 4, which is characterized by high expression of *Gfap*, and exhibits high expression of other DAA markers, such as *Gsn* and *Vim* (Figure 7B). Further, the gene expression profile associated with sub-cluster 4 is annotated to GO terms that reflect this potentially neurotoxic stress response, with terms such as “response to stimulus,” and “gliogenesis” (Figure 7C). Analysis of the changes in astrocyte distribution across each of the sub-clusters over time reveals a −48.88% change in the fraction of cluster 4 astrocytes 2 days post-FUS-BBBO (Figure 7D). At longer-term time-points the fraction of astrocytes belonging to this sub-cluster returns to baseline levels with only a −0.79% and −3.06% change from baseline at 4 and 7 days post-FUS-BBBO, respectively (Figure 7D). This acute decrease in the fraction of astrocytes in the DAA sub-cluster indicates a transient trend away from neurotoxic DAA, potentially in favor of protective phenotypes after FUS-BBBO exposure in hippocampal astrocytes.

### FUS-BBBO increases proportion of glioprotective astrocyte sub-populations

**Figure 8:**
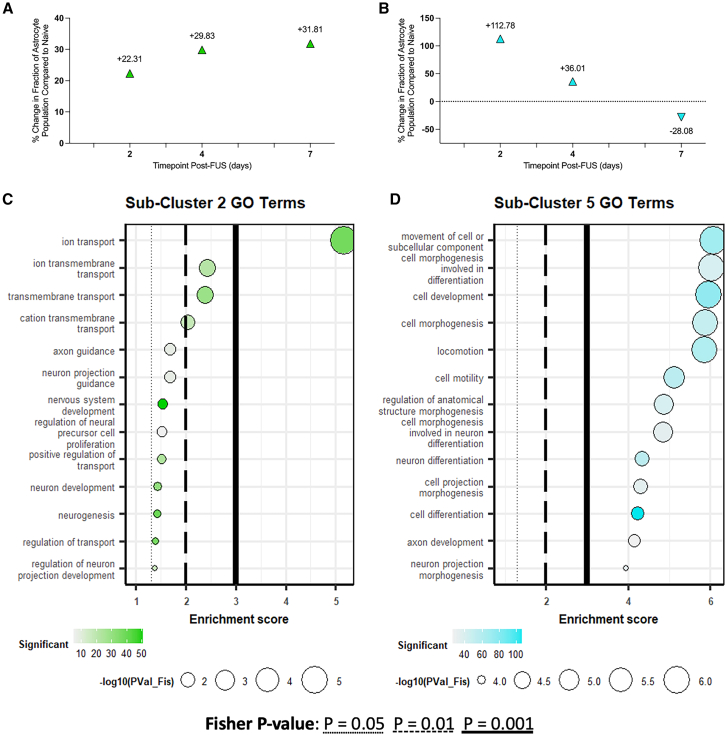
FUS-BBBO increases proportion of sub-clusters 2 and 5 astrocytes, promoting transport and neuronal development (A and B) The percent change in the fraction of astrocytes in sub-clusters 2 (A) and 5 (B) relative to naive are shown. (C) Sub-cluster 2 differential gene expression is annotated to gene ontology terms related to signaling and ion transport. (D) Sub-cluster 5 gene expression is annotated terms related to neuronal development and morphogenesis. Dot size represents the -log_10_(Fisher *p* value) and dot color represents the number of significant genes annotated to each term for (C) and (D).

Analysis of the changes in the percentage of astrocytes belonging to each sub-cluster over time after FUS-BBBO reveals a sustained increase up to 31.81% in sub-cluster 2 and a transient increase of sub-cluster 5 up to 112.78% 2 days post-FUS (Figures 8A and 8B). The early and sustained increase in sub-cluster 2 indicates that after FUS, the population of astrocytes evolves to adopt the sub-cluster 2 phenotype with increasing frequency. Given the neuroprotective genes upregulated in sub-cluster 2 relative to other sub-clusters, this points to a positive and neuroprotective evolution following FUS-BBBO in hippocampal astrocytes (Figure 6C). Further, GO analysis of the gene expression profile for sub-cluster 2 indicates a role in directing ion transport, signaling and neuronal development with terms, such as “cation transmembrane transport,” “nervous system development,” and “regulation of transport” (Figure 8C).

Sub-cluster 5 presents another sub-population that grows, albeit transiently, following FUS-BBBO exposure. In particular, the fraction of astrocytes in sub-cluster 5 increases by 112.78% 2 days after FUS-BBBO, before beginning to return to baseline levels at 4 and 7 days post-FUS-BBBO (Figure 8B). The gene signatures and GO annotations alike indicate that this sub-population of astrocytes is involved with active cellular component movement and promoting neuronal development with GO terms, such as “cell motility,” and “cell morphogenesis involved in neuron differentiation,” and “axon development” (Figure 8D). The transient increase in this population indicates that the increase in movement and cell motility is an acute response to FUS-BBBO, however overall this population appears to direct morphological changes and neuron development.

Taken together the increase in the prominence of sub-cluster 2 and 5 astrocytes following FUS-BBBO indicates a trend toward glioprotective phenotypes following FUS-BBBO.

### FUS-BBBO does not induce significantly different transcriptomic response between male and female WT hippocampal astrocytes

Male and female astrocytes were identified based on their expression of the *Xist* gene (Figure S4A). Differential gene expression analysis between male and female astrocytes at each time point after FUS-BBBO revealed very few significant differences. In fact, the only significant difference detected between male and female astrocytes at a given time-point was in the expression of *Gm47283*, 2 days post-FUS-BBBO (Figure S4B). Female expression of *Gm47283* was higher than male in naive astrocytes, as well as at 2 and 4 days post-FUS-BBBO, but significant only at 2 days post-FUS-BBBO (adjusted *p-*value = 0.0023, Wilcoxon rank-sum test). Other genes with nonsignificant differences in expression between males and females are shown in Figure S4. These include *Pla2g7*, *Gm26917*, and *Slc38a3*. Overall, very few differences were observed between male and female astrocytes after FUS-BBBO.

## Discussion

The dynamic response of astrocytes to FUS-BBBO as demonstrated by the significant up- and down-regulation of several genes at all three time points evaluated (2, 4 and 7 days) relative to naive controls indicates that there are both early and sustained effects in hippocampal astrocytes (Figure 2A). The time point with the greatest number of significant differentially expressed genes was at two days post-FUS-BBBO. However, 293 genes were significantly upregulated at all three time points, indicating a significant and sustained response in all FUS-treated cells evaluated here (Figure 2B).

Focusing on three of the most significant shared genes upregulated at these three time points in Figure 2C reveals interesting trends regarding the potential for FUS-BBBO to activate hippocampal astrocytes. Transforming growth factor betas (*Tgfb*s) are growth factors implicated in critical processes sustaining brain homeostasis. In particular, *Tgfb1* is has been identified as an important factor for promoting neurogenesis, synapse formation, and angiogenesis.^38^ The expression of the *Tgfb1* receptor, *Tgfbr1*, is necessary for transmembrane *Tgfb1* transmission, which may instruct synaptogenesis in adjacent neurons.^39^ The upregulation of this receptor at short- and long-term time-points after FUS-BBBO indicates a potential for increased astrocyte-mediated synaptogenesis following FUS-BBBO. The complement cascade, of which *C1qa* is a component, controls astrocytic synapse elimination, especially in mouse models of neurodegenerative disease.^40^ The upregulation of *C1qa* at all time-points post-FUS-BBBO suggests an increase in complement-mediated synapse elimination by hippocampal astrocytes. Finally, *Tmsb4x* is implicated in actin polymerization and cellular component movement, indicating a dynamic movement response in affected astrocytes with the upregulation of this gene at all time-points evaluated post-FUS-BBBO.

Although insufficient to completely describe the heterogeneous phenotypes of astrocyte populations *in vivo*, a binary classification system of A1 and A2 activations states offers a useful framework for categorizing phenotypic activation as neurotoxic or neuroprotective respectively.^3^ Common markers of reactive, A1 activated astrocytes include *Gfap*, *Vim*, *Serping1*, *Psmb8* and especially *C3*.^3^^,^^41^^,^^42^^,^^43^ Evaluation of the relative expression of these genes compared to naive animals reveals an upregulation of some (*Psmb8*), but notably a down-regulation in others (*Gfap*, Vim, Serping1, and *C3*). This observation, compared with the previous observation of extensive transcriptomic changes relative to controls raises the question of what kind of activation and processes these cells are undergoing, if not neurotoxic. Thus, the relative expression levels of A2 neuroprotective markers *Stat3*, *S100a10*, and *Cd14* were evaluated.^3^ The observed upregulation of two of these markers at both acute and long-term time points after FUS-BBBO, *Stat3* and *Cd14*, provides a positive indication of a shift toward this activation state (Figure 3A).

Severe astrogliosis occurring in response to injury is characterized by astrocytic proliferation and migration to the site of insult, where a glial scar may form.^3^ This extreme form of neurotoxic astrocytic activation is associated with loss of homeostatic astrocytic function and broadly considered a negative physiological response. An increase in proportion of astrocytes measured within an affected area would indicate that perhaps glial scarring and astrocytic proliferation were occurring and extreme gliosis was occurring. Favorably, when measuring the fraction of ACSA-II+ astrocytes at short- and long-term time points following FUS-BBBO, we found no significant differences in the proportion of astrocytes in FUS-treated hippocampal tissue compared with anesthesia controls (Figures 3B–3E). This held true for both male (Figure 3B) and female (Figure 3C) aged (1 year-old) WT animals, and young (Figure 3D) and aged (Figure 3E) 3xTg-AD females. Taken together, these findings indicate that astrocytic activation in response to FUS-BBBO with the parameters used in this study is dominated by glioprotective gene signatures and does not induce reactive gliosis and subsequent glial scarring. This finding is consistent with previous reports finding no significant astrocytic proliferation in response to similar FUS-BBBO parameters.^44^

Astrocytes perform essential functions at neuronal synapses, participating as the third component of tripartite synapses with pre- and post-synaptic neurons.^45^ In addition to their supportive role moderating efficient signaling by regulating neurotransmitter concentration and synapse health, astrocytes are believed to also play a role in directing communication by actively modifying synapses and modulating their plasticity.^45^ In response to FUS-BBBO, hippocampal astrocytes exhibit gene expression changes annotated significantly to many terms related to synaptic maintenance and modification. Terms such as “neuron to neuron synapse,” “synapse organization,” and “regulation of synapse organization,” suggest that these astrocytes are participating actively in synapse modification (Figure 4A). Interestingly, annotation to synapse terms is most significant 2 days post-FUS-BBBO, with waning significance at 4 and 7 days post-FUS-BBBO. This observation indicates that FUS-BBBO may trigger an acute and transient boost in synapse modification and optimization, becoming less prominent with time after treatment.

The movement of astrocytic processes is at times necessary to perform the aforementioned synaptic maintenance functions, in addition to performing other glioprotective roles. It has been shown that astrocyte processes can move rapidly to contact dendritic spines and affect neuronal synapses.^46^ Astrocytic process extension may also provide a scaffold for the growth or regeneration of neurons.^47^ The observed upregulation in genes annotated to astrocytic process extension therefore may have various diverse implications, many of which have the potential to both actively promote neuronal health and functioning, in addition to offering trophic support. Thus, the gene expression changes in FUS-treated astrocytes that are significantly annotated to terms, such as “regulation of cell projection organization,” “regulation of cellular component movement,” and “anatomical structure formation involved in morphogenesis,” indicate that FUS-BBBO may affect these processes. Unlike the terms related to synaptic support, annotations to cellular component movement terms increase in significance with time after FUS-BBBO, suggesting that cellular component movement may be a delayed and sustained response to FUS-BBBO (Figure 4B).

*Wnt3* is one of the most prominent Wnt proteins implicated in adult hippocampal neurogenesis, making the high upregulation at 2 days post-FUS relative to untreated control astrocytes a promising indication of the potential for FUS-activated astrocytes to promote neurogenesis.^48^ Thus, the observed increase not only in the expression level, but in the percentage of astrocytes expressing *Wnt3* indicates that affected astrocytes may be promoting neurogenesis, confirming findings by our group and others.^21^^,^^28^^,^^29^^,^^44^ Further, *Il6* and *Il1b* are additional factors implicated in adult neurogenesis.^16^ More specifically, these factors promote differentiation of progenitor cells to mature neurons, which may be protective and therapeutic, especially in the context of neurodegenerative disease. *Il6* and *Il1b* are both upregulated in a small fraction of the astrocytes analyzed here, at long-term time points after FUS-BBBO, lending further support to the hypothesized role of astrocytes in promoting adult neurogenesis (Figure 5A). An increase in GABAergic or glutamatergic neurons has been observed resulting from increased astrocytic expression of *Ascl1*, making this a potential neurogenesis promotional factor of interest.^49^ These data reveal a peak in *Ascl1* expression 4 days post-FUS-BBBO relative to naive controls. Finally, fibroblast growth factor 2, encoded by *Fgf2* and also upregulated relative to controls following FUS-BBBO induces proliferation of neuronal stem cells, and thus may promote neuronal health and resilience in a neurodegenerative model.^16^

Stimulating adult neurogenesis is of great interest for sustaining neuronal health and promoting regeneration in neurodegenerative disease. It has been well documented that FUS-BBBO is capable of increasing neurogenesis in the adult murine brain.^21^^,^^28^^,^^29^^,^^44^ Still, the mechanism by which this has been achieved remains insufficiently characterized. The data presented here proposes a role for astrocytes in stimulating and facilitating this process. Neurogenesis is of interest not only for its promotion of neuronal network health on a cellular, microenvironmental level, but also for its implications in cognition and neuropsychiatric symptoms. It has been suggested not only that hippocampal neurogenesis is capable of exerting anxiolytic effects but also that astrocytes are critical players in mediating this effect.^50^^,^^51^ This may retroactively explain some of the anti-anxiety effects observed following FUS-BBBO exposure alone in previous works.^25^^,^^52^ In particular, it has been shown that early, repeated intervention with FUS-BBBO is capable of preventing the progression of disease-associated anxiety in 3xTg AD mice.^25^ Further, it has been observed that adult neurogenesis declines with age and AD progression, perhaps contributing to age- and AD-associated cognitive decline.^53^ Thus, a FUS-induced, astrocyte-mediated boost in adult neurogenesis may confer neuroprotective effects with far-reaching implications in anxiety, neuronal health and cognition. This boost may be responsible for the improvements in AD-associated spatial memory and reversal learning observed in murine AD models after FUS-BBBO in both an early, prevention and late-stage therapeutic intervention paradigms.^23^^,^^25^^,^^54^ Previous studies have elucidated a role for FUS-BBBO in spatial memory improvement in AD mice relative to untreated controls. This improvement may be a result of improved synaptic strength and long-term potentiation, which subsequently improves hippocampal plasticity.^54^ The data presented herein indicate that astrocytes may play a key role in enacting these benefits.

Astrocytes contact and communicate with many different cell types as members of both tripartite synapses and the BBB. Of particular interest to neuronal health and neurodegenerative disease is their connection and communication with oligodendrocytes, the myelinating cells of the CNS. Astrocytes and oligodendrocytes are connected by gap junction protein complexes, facilitating and evidencing the importance of their direct contact and communication.^55^ Astrocytic signaling to oligodendrocyte precursor cells (OPCs) and oligodendrocytes is critical for coordination of myelination and repair, in addition to maintenance of homeostasis and neuronal health.^56^ This communication may occur via secreted signaling molecules or directly via gap junctions formed between astrocytes and oligodendrocytes.^56^ Specifically, astrocytes are capable of promoting oligodendrocyte proliferation, maturation and survival via secreted factors such as platelet-derived growth factor (*Pdgf*), fibroblast growth factor (*Fgf*), and leukemia inhibitory factor (*Lif*).^57^^,^^58^ Tissue inhibitors of metalloproteinases (*Timp*s) are extracellular proteins expressed on astrocytes that affect OPCs through receptor-mediated signaling.^59^ In particular, astrocytic *Timp1* expression has been identified as a critical factor in promoting OPC maturation and myelination *in vivo*.^59^^,^^60^ Finally, astrocytic engagement of the nuclear factor erythroid-2-related factor-2 (Nrf2) pathway, encoded by the *Nfe2l2* gene, has been identified as a pro-myelination pathway that also confers neuroprotective benefits to surrounding neurons.^61^^,^^62^ Interestingly, our data reveal an increase in the expression of all of these factors after FUS-BBBO exposure (Figure 5). *Lif* expression increases at 2 days post-FUS-BBBO, returning to baseline at longer-term time points. Similarly, *Timp1* and *Igf1*, which are expressed in only a small fraction of astrocytes, exhibit peak expression 2 days post-FUS-BBBO, indicating an acute response to FUS-BBBO in astrocytic signaling to oligodendrocytes and OPCs. *Pdgfb* and *Nfe2l2* are expressed by a greater fraction of the hippocampal astrocytes studied here, and we observe a sustained upregulation in both genes across all three time points measured post-FUS-BBBO compared to naive controls. This both short- and long-term upregulation offers a positive indication for longer-term astrocytic support of OPC differentiation and oligodendrocyte growth after FUS-BBBO. Finally, although categorized in Figure 5 as a neurogenesis-promoting factor, *Fgf2* has been linked to both processes, making the observed increase a promising indication of astrocyte-directed promotion of multiple glioprotective processes.

Myelinating oligodendrocyte glycoprotein (MOG) is a marker of mature, myelinating oligodendrocytes. The observed trending increase in the percentage of MOG+ cells after treatment indicates that FUS-BBBO may be capable of inducing OPC differentiation and promoting myelination, perhaps as a consequence of astrocytic signaling as previously discussed. Notably, the increase in the percent of MOG+ cells two days after FUS-BBBO is more than twice as extensive in 10-week old (+102.3%) as in 1-year-old 3xTg (+48.4%) groups. Further, the increase in MOG+ oligodendrocytes is sustained at 7 days in 10-month-old 3xTg females (+99.7% compared to 7-day sham group), but not in 1-year-old 3xTgs (−16.9% compared to 7-day sham group). This discrepancy is consistent with expectations given that the rate of OPC differentiation slows with the progression of age and that AD likely disrupts OPCs, thereby preventing efficient differentiation and myelination.^63^^,^^64^ This finding indicates that younger, asymptomatic AD brains may stand to benefit most from FUS-BBBO in terms of the regenerative capacity of their native OPCs and myelinating mechanisms, potentially resulting from astrocytic activation.

The astrocyte sub-clusters analyzed here represent a heterogeneous astrocyte population with diverse functions. The composition of hippocampal astrocytes evolves over time after FUS-BBBO, with the percentage of astrocytes belonging to each sub-population changing over time (Figure 6B). While the response profile of astrocytes varies in part due to their inherent heterogeneity, another factor that likely contributes to the observed differences between sub-clusters may be their spatially inhomogeneous exposure to FUS-BBBO in the paradigm evaluated herein. FUS-BBBO is a targeted intervention that most significantly affects cells and tissue within the focus, meaning that cells adjacent to or outside of the focus may be affected or activated to a lesser extent. Silburt and Aubert developed a technique for identifying astrocytes that are proximal and focal to an activating stimulus based on morphological features and their expression of GFAP and Nestin.^65^ This technique elucidates the observable morphological differences between astrocytes near to, and far from the FUS focus 7 days after exposure, which would likely exhibit diverging transcriptomic signatures and appear in different astrocyte sub-clusters. Thus, the sub-clusters observed in the present study may represent astrocytes at varying spatial positions relative to the FUS focus in the hippocampus. Spatial transcriptomics overlaid on histological images would likely serve as a helpful link to map astrocyte sub-clusters on to the proximal or focal populations described by Silburt and Aubert.

One of the most significantly upregulated genes by astrocytes in cluster 0 is *Elmo1*, which is believed to participate in a phagocytosis pathway associated with reactive astrocytes. Cluster 1 exhibits high expression of *Actb* relative to other sub-clusters, which is a cytoskeletal component contributing to cell movement and morphology, but is also expressed at very stable levels, making it a commonly used housekeeping gene. Cluster 2 exhibits prominent upregulation of metallothionein 1 and 3 (*Mt1* and *Mt3*), which have been identified to be protective in the context of neurodegenerative disease and CNS insult.^66^ Cluster 3 is characterized by an increased expression of synaptic nuclear envelope protein 1 (*Syne1*) and microtubule actin cross-linking factor 1 (*Macf1*). Interestingly, cluster 4 exhibits high expression of *Gfap*, designating it as the reactive, “*Gfap*-high” cluster in tis heterogeneous astrocyte population. Finally, cluster 5 is characterized by high expression of gap junction protein gamma 1 (*Gjc1*) indicating increased communication via gap junctions, which may be between adjacent astrocytes or between astrocyte and oligodendrocytes.

The majority of the astrocytes studied in each time-point group belong to sub-clusters 0 and 1. The percentage of astrocytes in these sub-clusters remains relatively stable before and after FUS-BBBO and likely represents homeostatic astrocytes performing normal maintenance functions. GO analysis of these two sub-clusters is dominated by terms such as “regulation of biological quality” and “homeostatic process” further demonstrating their stable, homeostatic role (data not shown). The dynamic changes in the prominence of smaller sub-clusters after FUS-BBBO, elucidate the effects of FUS-BBBO on hippocampal astrocytes over time.

The upregulation of *Mt1* and *Mt3*, as well as the downregulation of *Matalat1*, a gene sometimes correlated with reactive, neurotoxic astrocytes, nominates cluster 2 as a neuroprotective sub-population of astrocytes (Figure 6C). West et al. identify a protective role for metallothionein gene expression in astrocytes in pathological conditions of CNS disease.^66^ In addition to their protective role on a cellular level in neurodegenerative disease, they also mitigate mercury neurotoxicity and may directly influence higher-level cognition.^66^ *Mt3* contributes to actin polymerization and subsequent amyloid clearance by astrocytes, and is downregulated in AD.^67^ Further, Lee et al. identify that the absence of *Mt3* stunts astrocytic amyloid-β clearance, further evidencing its role in facilitating amyloid clearance and promoting neuroprotection in AD.^67^ Cluster 2 increases in prominence at 2, 4, and 7 days post-FUS-BBBO, indicating that this intervention shifts the heterogeneous distribution of astrocytes in favor of a protective phenotype (Figure 8A). In addition to the upregulation of known neuroprotective genes by this sub-population of astrocytes, GO analysis of the gene expression profile of this sub-cluster consists of several terms associated with signaling and neuronal development such as “transmembrane transport” and “nervous system development.” It has been observed that increasing intracellular calcium in hippocampal astrocytes may give rise to anxiolytic effects.^51^ Thus the prominence of GO terms such as “ion transmembrane transport” and “positive regulation of transport” by sub-cluster 2 suggest a potential role for these hippocampal astrocytes in facilitating anti-anxiety behaviors following FUS-BBBO (Figure 8C). Collectively, these observations may promote FUS-BBBO as a promising intervention that may activate astrocytes to exhibit anxiolytic effects, improve cognition, and improve Aβ clearance.

Sub-cluster 5 is characterized by increased expression of genes related to signaling such as *Gjc1* and *Slco3a1*, and decreased expression of potentially harmful genes such as *Cst3* and *Apoe* (Figure 6C). Notably, the fraction of astrocytes belonging to this sub-cluster increases at 2 and 4 days post-FUS-BBBO, indicating a push toward signaling and protective phenotypes (Figure 8B). Further, cluster 5’s gene expression profile is annotated to terms such as “cell development,” “cell motility,” and “neuronal differentiation,” indicating a role for this population in facilitating neuronal development, which may enhance neuronal health and functioning, and subsequently supporting cognitive function. Enhanced neuronal support is particularly beneficial in pathological conditions of neurodegenerative disease where neuronal circuit activation is required for regeneration and development.

These observations may begin to retroactively explain the many studies that report improvement in anxiety, cognition, and pathological accumulation with the application of FUS-BBBO alone in murine models of AD.^23^^,^^25^^,^^26^^,^^27^ The interaction and coordination of multiple cell types are certainly at play to facilitate the preventative and therapeutic benefits observed in these studies following FUS-BBBO application, however these data indicate that astrocytes may play an especially prominent and critical role.

The discovery of a transcriptomic signature associated with age and AD in WT and 3xTg mice respectively by Habib et al. has enabled deeper understanding of sub-populations of astrocytes exhibiting high expression of these markers.^13^ In particular, our data reveal that sub-cluster 4 represents the population of astrocytes most closely resembling these DAA, and therefore evaluation of the change in the fraction of astrocytes belonging to this cluster over time after exposure to FUS-BBBO enables an understanding of the way FUS-BBBO may influence its prominence. Our data indicate that the fraction of astrocytes belonging to this DAA clusters reduces transiently, at 2 days post-FUS-BBBO, and is restored to baseline at 4 and 7 days post-FUS-BBBO (Figure 7C). These findings suggest that FUS-BBBO may give rise to an acute reduction in the prominence of this neurotoxic, disease-associated population. Interestingly, the return to baseline prominence 4 days post-FUS-BBBO represents a time point after the expected time for BBB reinstatement at the FUS parameters used. Perhaps there is a relationship between the timeline of BBB reinstatement and the observed decrease in DAA prominence, indicating that this effect may be confined to a short time frame post-FUS-BBBO. The parallel increases in sub-clusters 2 and 5 after FUS-BBBO, with gene signatures annotated to neuroprotective GO terms, indicates that a reduction in DAA may occur in favor of an increase in responsive sub-populations that promote neuronal health and development, rather than detract from it.

The early and sustained increase in sub-cluster 2 astrocytes following FUS-BBBO indicates a persistent shift in the astrocytic population after FUS-BBBO (Figure 8A). The GO terms annotated to sub-cluster 2 genes relate mostly to ion transport and nervous system development, indicating that a greater proportion of astrocytes may be allocated for these processes after FUS-BBBO than in naive animals.

The transient increase in the proportion of sub-cluster 5 astrocytes after FUS-BBBO indicates a population shift toward their phenotype and homeostatic role (Figure 8B). The gene expression profile differentiating sub-cluster 5 from other astrocyte sub-populations studied here reveals GO annotations related largely to cellular movement and neuronal development (Figure 8D). There are several processes enabled by astrocytic morphogenesis and process movement. One such function is the extension of PAPs. The extent of PAP synapse coverage, which varies with time and in response to stimuli, is mediated by actin-dependent motility.^6^ The dynamic nature of astrocytic postsynaptic density (PSD) has been extensively documented, especially in response to changes in synaptic firing and external stimuli.^6^^,^^68^ Thus, our observation of upregulated GO terms related to morphogenesis and locomotion is unsurprising given that these astrocytes are expected to modify their structure in response to stimuli. The increase in GO terms annotated to cell motility indicates that perhaps, with time after FUS-BBBO, hippocampal astrocytes may extend their synapstic coverage in order to more extensively cover the synapse to regulate glutamate uptake. Astrocyte morphogenesis and process ramification is also expected to contribute to synaptogenesis and maintenance, neurotransmitter recycling, synapse plasticity modulation and BBB maintenance.^69^ In the case of FUS-BBBO, this likely also extends to BBB reinstatement, which is required only transiently after FUS-BBBO. The expected BBB reinstatement timeline for the parameters used here is consistent with the acute increase in sub-cluster 5 astrocytes. Perhaps, a subset of astrocytes adopt a restorative phenotype in response to BBBO, and give way to other sub-clusters and phenotypes once the barrier is restored.

The disparity between female and male incidence of neurodegenerative disease, particularly AD, has raised interest in evaluating sex as a biological variable. To this end, we included both female and male WT mice in the present transcriptomic study to evaluate any differences that may emerge in their response to FUS-BBBO, a proposed therapeutic for neurodegenerative disease, to better predict any differences in response that may be observed in clinical translation. Interestingly, our data reveal no significant differences between the gene expression profiles of male and female astrocytes at each time point post-FUS-BBBO outside of sex-linked genes that are reflected also in the comparison of naive male and female astrocytes (Figure S4). This offers promising support for FUS-BBBO as an unbiased, gender-independent treatment of male and females, at least in terms of their astrocytic activation, with FUS-BBBO.

The data presented herein elucidate the role of astrocytes in directing cellular-level responses to FUS-BBBO in the hippocampus of aged, WT, and AD mice. The findings presented herein indicate that FUS-BBBO activates hippocampal astrocytes toward a glioprotective state, promoting transient synapse modification and a sustained increase in cellular component movement that may confer protective benefits for making neural circuits more efficient, all without promoting significant gliosis in aged WT and young and aged 3xTg-AD mice alike. Further, FUS-BBBO promotes astrocytic signaling that may promote neurogenesis and OPC differentiation, facilitating the generation of new neurons as well as increasing the potential for myelination. All of these protective effects are observed in parallel with a decrease in sub-populations resembling neurotoxic, disease-associated astrocytes following FUS-BBBO exposure. Taken together these data reveal a glioprotective role for astrocytes following FUS-BBBO that may retroactively explain the improvement in cognition, anxiety, and pathology in murine models of AD, strengthening our understanding of the bioeffects of FUS-BBBO as clinical translation and adoption become more widespread.

### Limitations of the study

This study offers in-depth transcriptomic characterization of the astrocytic response to FUS-BBBO over time. The observed changes in gene expression and sub-population dynamics provide the first pieces of evidence necessary for validating the role of astrocytes in complex processes such as neuronal development and cognition. Measurement with FACS provides further support for these claims. However, future behavioral experiments should be conducted to fully validate the hypothesized roles described in the present study. Additionally, the entire hippocampus was dissected for single-cell RNA sequencing and FACS experiments, however FUS exposure was not necessarily uniform across all hippocampal astrocytes, as discussed in the discussion section.

## Resource availability

### Lead contact

Further information and requests for resources and reagents should be directed to and will be fulfilled by the lead contact, Elisa E. Konofagou (ek2191@columbia.edu).

### Materials availability

This study did not generate new unique reagents.

### Data and code availability

• Data: Single-cell RNA-seq data have been deposited at NCBI GEO and are publicly available as of the date of publication. Accession numbers are listed in the key resources table.
• Code: Sequencing analysis was performed using standard RNA sequencing pipelines in R using the Seurat package^70^ and TopGO.^71^
• Additional information: Any additional information required to reanalyze the data reported in this paper is available from the lead contact upon request.

## Acknowledgments

The authors would like to thank Michael Kissner BA and Juan Idiarte Montiel BS for their extensive support and contributions to optimizing the flow cytometry components of the present study. We would also like to thank Vilas Menon PhD for his support and guidance in establishing the single-cell sequencing analysis pipeline used for the present study. This work was supported by the 10.13039/100000002National Institutes of Health Grant R01AG038961 (E.E.K.), 10.13039/100000002National Institutes of Health Grant R01EB029338 (E.E.K.), and National Science Foundation Graduate Research Fellowship (R.L.N.).

## Author contributions

R.L.N., A.R.K.-S., and E.E.K. conceptualized the single-cell sequencing experiments. R.L.N. and E.E.K. conceptualized the flow cytometry experiments and R.L.N. performed the FUS-BBBO treatments. R.L.N. and A.R.K.-S. performed single-cell sequencing preparation. A.J.B., A.R.K.-S., N.K., and F.T. performed cardiac perfusions. A.R.K.-S. performed sequencing alignment and hashtag demultiplexing. R.L.N. performed differential gene expression and subsequent sequencing analysis. R.L.N. wrote the manuscript with support from E.E.K. and A.J.B. All contributing authors have approved the present manuscript.

## Declaration of interests

Some of the work presented herein is supported by patents licensed to Delsona Therapeutics, Inc. where E.E.K. serves as co-founder and scientific adviser.

## STAR★Methods

### Key resources table

**Table:** 

REAGENT or RESOURCE	SOURCE	IDENTIFIER
**Antibodies**		

ACSA-II-PE conjugated antibody	Miltenyi	#130-123-284; RRID: AB_2811488
TotalSeq™-B0301 anti-mouse Hashtag 1	BioLegend	#155831; RRID: AB_2814067
TotalSeq™-B0302 anti-mouse Hashtag 2	BioLegend	#155833; RRID: AB_2814068
TotalSeq™-B0303 anti-mouse Hashtag 3	BioLegend	#155835; RRID: AB_2814069
TotalSeq™-B0304 anti-mouse Hashtag 4	BioLegend	#155837; RRID: AB_2814070
TotalSeq™-B0305 anti-mouse Hashtag 5	BioLegend	#155839; RRID: AB_2814071
TotalSeq™-B0306 anti-mouse Hashtag 6	BioLegend	#155841; RRID: AB_2814072
TotalSeq™-B0307 anti-mouse Hashtag 7	BioLegend	#155843; RRID: AB_2814073
TotalSeq™-B0308 anti-mouse Hashtag 8	BioLegend	#155845; RRID: AB_2814074
Alexa Fluor® 488 Anti-Myelin oligodendrocyte glycoprotein antibody	Abcam	ab306602

**Chemicals, peptides, and recombinant proteins**		

Gadomide contrast agent	Omniscan, GE Healthcare	N/A

**Critical commercial assays**		

Neuron Neural Tissue Dissociation Kit	Miltenyi	# 130-094-802
MACS buffer	Miltenyi	#130-091-376
Myelin II removal beads	Miltenyi	#130-096-433
LS columns	Miltenyi	#130-042-401

**Deposited data**		

Raw and analyzed data	This paper	NCBI GEO: GSE274108

**Experimental models: Organisms/strains**		

Mouse: B6.129P2(Cg)-Cx3cr1tm1Litt/J	The Jackson Laboratory	Strain #:005582
Mouse: B6; 129-Tg(APPSwe, tauP301L)1Lfa Psen1tm1Mpm/Mmjax	The Jackson Laboratory	MMRRC Strain #034830-JAX

**Software and algorithms**		

CellRanger	10x Genomics	Cellranger-7.0.0
Seurat: R toolkit for single cell genomics	Hao et al.^70^	N/A
topGO Enrichment Analysis for Gene Ontology	Alexa and Rahnenfuhrer^71^	N/A
FlowJo	FlowJo.com	N/A
GraphPad Prism		

**Other**		

Vybrant DyeCycle Ruby	Invitrogen	#V10309

### Experimental model and study participant details

#### Animal use

All animals were housed and handled in compliance with Columbia University’s Institutional Animal Care and Use Committee under protocol #AC-AABG4559. A total of 16 male and 16 female 10-week-old C57BL background mice were used for single-cell RNA sequencing experiments (Figure 1A). The animals were treated in four cohorts, with one male and one female in each timepoint group (naive, 2 days, 4 days or 7 days post-FUS-BBBO) per group for single-cell sequencing preparation and analysis, resulting in a total of 4 male and 4 female mice for each timepoint condition. The experiment was designed in this way to include all conditions (experimental timepoint, sham, and sex) to mitigate batch effects. 12 male and 12 female 1-year-old C57BL background mice were used for flow cytometry analysis of astrocyte proliferation (Figure 3B and 3C). 18 10-week-old and 18 1-year-old female 3xTg-AD, female mice were used for flow cytometry analysis of astrocyte and oligodendrocyte populations (Figures 3D, 3E, and 5C–5E).^72^ For flow cytometry experiments mice were administered bilateral hippocampal FUS-BBBO or anesthesia, and sacrificed either 2 or 7 days later for flow cytometry analysis.

### Method details

#### Study design

This study was designed to elucidate the transcriptomic changes in hippocampal astrocytes at long- and short-term timepoints after FUS-BBBO exposure. The safety of the treatment paradigm and FUS parameters used here are well-established.^73^ Single-cell RNA sequencing was used to evaluate the transcriptomic changes in hippocampal astrocytes in male and female, 10-week-old WT mice after FUS-BBBO. Flow cytometry was used to evaluate astrocytic and oligodendrocyte populations in aged WT and young and aged AD mice. In total this study was intended to clarify the nature and persistence of the response to FUS-BBBO in hippocampal astrocytes.

#### Focused ultrasound blood-brain barrier opening

Focused ultrasound-induced blood-brain barrier opening (FUS-BBBO) was performed using safe, well-established parameters.^73^ A single-element, concave FUS transducer (center frequency: 1.5 MHz, focal length: 60 mm, diameter: 60 mm; Imasonic, France) was operated at a Peak Negative Pressure (PNP) of 450 kPa with a pulse repetition frequency of 10 Hz and pulse length of 10,000 cycles. An additional single-element transducer (V320, frequency: 7.5 MHz, focal length: 52 mm, diameter: 13 mm; Olympus NDT, Waltham, MA, USA) was confocally aligned with the FUS transducer focus and used for passive cavitation detection to monitor microbubble activity in real-time during sonication. The transducers were fixed to a 3D positioning system for targeting. The FUS setup is shown in Figure 1B. For each BBBO session the mouse was anesthetized with 3–4% isoflurane until induction was confirmed by lack of response to toe pinch. Mouse heads were stabilized using a stereotactic apparatus and anesthesia was maintained via a nose cone delivering 1.5–2% isoflurane for the duration of the session. Each mouse’s head was shaved with electric clippers, then residual hair was removed with depilatory cream and subsequently covered with degassed ultrasound coupling gel and a bath of degassed water. The transducer was submerged in the water bath and targeting was performed to center the transducer over the lambdoid structure. 10-s control pulses were then recorded at each sonication target to quantify baseline cavitation in the absence of microbubbles. Next, a 50-μL bolus of in-house made, lipid-shelled, polydisperse microbubbles at a concentration of 8x10^8^ microbubbles/μL was intravenously injected and the transducer was moved to sonicate each target location for 60 s while microbubble activity was recorded in real time. For single-cell RNA sequencing experiments two brain regions were targeted for BBBO, one per bilateral hippocampus located 2 mm rostral and 1.8 mm lateral to the lambdoid structure (Figure 1C). For flow cytometry experiments, four targets were sonicated with two targets per bilateral hippocampus. These targets were located 1.5 mm rostral and 2.5 mm lateral, then 2.5 mm rostral and 1.5 mm lateral to the lambdoid structure on each side, with a bolus injection of microbubbles injected before the first and third target spot.

#### Magnetic resonance imaging

Following FUS-BBBO a T1-weighted magnetic resonance image was acquired for each mouse to for *in vivo* BBBO and targeting confirmation. A 0.2 mL intraperitoneal injection of gadomide (Gd) contrast agent (Omniscan, GE Healthcare, Chicago, IL, USA) was administered to each animal and allowed to diffuse for 15 min while the mouse was anesthetized with isoflurane. Contrast-enhanced MR images were then acquired for each mouse using a T1-weighted 2D FLASH sequence (Bruker Ascend 400 MHZ WB 9.4T, TR: 230 ms, TE: 3.3 ms, Flip angle: 70°, Averages: 6, FOV: 25.6 mm × 25.6 mm, Matrix size: 256 × 256, Slice thickness: 0.4 mm, Resolution: 0.1 mm × 0.1 mm, Scan time: 5 min). Axial and coronal images were acquired to confirm targeting and visualize the BBBO region *in vivo*.

#### Single-cell RNA sequencing

At the appropriate timepoint post-FUS-BBBO (2, 4 or 7 days) animals were euthanized with naive control animals by cardiac perfusion with sterile saline. The hippocampi of each animal were dissected out and timepoint-matched male and female tissue was pooled. The tissue was minced with a sterile razor and underwent enzymatic digestion according to the manufacturer’s protocol using a Postnatal Neuron Neural Tissue Dissociation Kit (Miltenyi, #130-094-802). Following enzymatic digestion the tissue was filtered using a 70-μm cell strainer and pelleted by centrifuging at 300xG for 10 min at room temperature. The pellet was then resuspended in 900 μL MACS buffer (Miltenyi, #130-091-376), and incubated with 100 μL Myelin II removal beads (Miltenyi, #130-096-433) for 15 min at 4°C, flicking to mix the solution every 5 min. The cells were pelleted at 4°C by centrifuging for 1 min at 9,300 RCF. The supernatant was discarded, and the pellet was resuspended in 1 mL of MACS buffer. This cell suspension was then passed through LS columns (Miltenyi, #130-042-401) on a magnet to extract the magnetic myelin beads. The cells were pelleted again for 1 min at 9,300 RCF, and resuspended in staining buffer, consisting of a 1:100 dilution of ACSA-II-PE conjugated antibody (Miltenyi, #130-123-284) and a 1:500 dilution of a unique hash tag antibody (Biolegend, #155801-8) for each timepoint group in 1xPBS. Cells were incubated with this staining solution for 30 min at 4°C. The cells were then pelleted, the supernatant was discarded, and the cells were resuspended in flow cytometry buffer consisting of a 1:1000 dilution of DAPI, 1:1000 Vybrant DyeCycle Ruby (Invitrogen, #V10309) and 1:500 DNase (from 4 mg/mL stock) in 1x PBS. The cells were then taken to the Columbia Stem Cell Initiative Flow Cytometry Core and live, ACSA-II+ astrocytes were sorted for each condition. These sorted cells were then brought to the Columbia Genome Center for single-cell RNA sequencing on a 10x Genomics Chromium Single Cell 3′ flow cell. The mean reads per cell varied across the sequencing runs analyzed in the present study, ranging on the order of 30,000–80,000 mean reads per cell.

ACSA-II, or anti-astrocyte cell surface antigen-II, is a selective surface marker of astrocytes.^74^ Selection of a surface marker for FACS was critical to enable sorting of live cells. ACSA-II was selected and used for FACS due to its robust labeling of hippocampal astrocytes and its insensitivity to papain-based tissue dissociation, which was used in to present study to prepare cells for sorting.^74^

#### Flow cytometry

The animals treated for flow cytometry analysis were euthanized by cardiac perfusion with sterile saline either 2 or 7 days post-FUS-BBBO or anesthesia exposure depending on their experimental group. The hippocampi of each animal were dissected out and processed independently, at which point tissue digestion and cell dissociation was conducted exactly as described above with two exceptions. First, the staining solution consisted of ACSA-II-PE astrocytic marker (Miltenyi #130-123-284) and anti-myelin oligodendrocyte glycoprotein (MOG) AF488 (Abcam, #ab306602) to evaluate the astrocyte and mature, myelinating oligodendrocyte populations simultaneously. Second, the live ACSA-II+ astrocytes and MOG+ oligodendrocytes were sorted and analyzed, with no subsequent RNA sequencing performed.

### Quantification and statistical analysis

#### RNA sequencing analysis

The raw sequencing data was aligned to the GRCm38 C57BL/6J mouse reference genome (mm10-2020-A) and de-multiplexing of the hashtag sequences was performed using Cell Ranger (cellranger-7.0.0). Subsequent analysis was performed in R using open-source packages from Seurat and topGO for differential gene expression and gene ontology (GO) analysis respectively.^70^ Integration and normalization was performed to cluster cells according to differential gene expression, enabling identification of various cell types based on canonical markers (Figures 1D, 1F, S1A, and S1B). Microglia were also isolated from the same murine samples used in the present study. These microglia were sorted and sequenced separately, but combined with astrocyte samples for the first QC steps of sequencing analysis, and subsequently appear in Figure 1D. All cells underwent quality filtering, removing cells with greater than 10% ribosomal and 15% mitochondrial RNA measured (Figures S2 and S3). Astrocytes were then selected out from the cells based on their expression of canonical markers, reintegrated, normalized, and used for all subsequent analysis. Nonlinear dimensional reduction was used to identify astrocyte sub-clusters (Figures 1E and 1G). Importantly, Figures S1C and S1D demonstrate an unbiased distribution of original run identity and treatment condition across the clusters.

### Statistical analysis

Differential gene expression was evaluated and significance was determined by Wilcoxon Rank-Sum test (Figure 2C). The -log_10_(Fisher *p*-value) is reported for GO results, and the number of significant genes annotated to each term is indicated by the color bar (Figures 4, 8C, and 8D). Pairwise comparisons for flow cytometry analysis were performed using unpaired t tests in Graphad Prism (Figures 3B–3E, 5C, and 5D). Significance is indicated throughout as follows: ∗*p* ≤ 0.05, ∗∗*p* ≤ 0.01, ∗∗∗*p* ≤ 0.001, ∗∗∗∗*p* ≤ 0.0001.
