# Supplementary material for: Focused ultrasound-induced blood-brain barrier opening promotes glioprotective phenotypes in ACSA-II+ murine astrocytes
Source: iScience. 2025 Jul 22;28(8):113173. doi: 10.1016/j.isci.2025.113173 (PMC12432449; doi:10.1016/j.isci.2025.113173)
Supplement: Document S1. Figures S1–S4 and Table S1 [file mmc1.pdf]

iScience, Volume *28*

Supplemental information

Focused ultrasound-induced blood-brain barrier opening promotes glioprotective phenotypes in ACSA-II+ murine astrocytes

Rebecca L. Noel, Alina R. Kline-Schoder, Alec J. Batts, Nancy Kwon, Fotios Tsitsos, and Elisa E. Konofagou

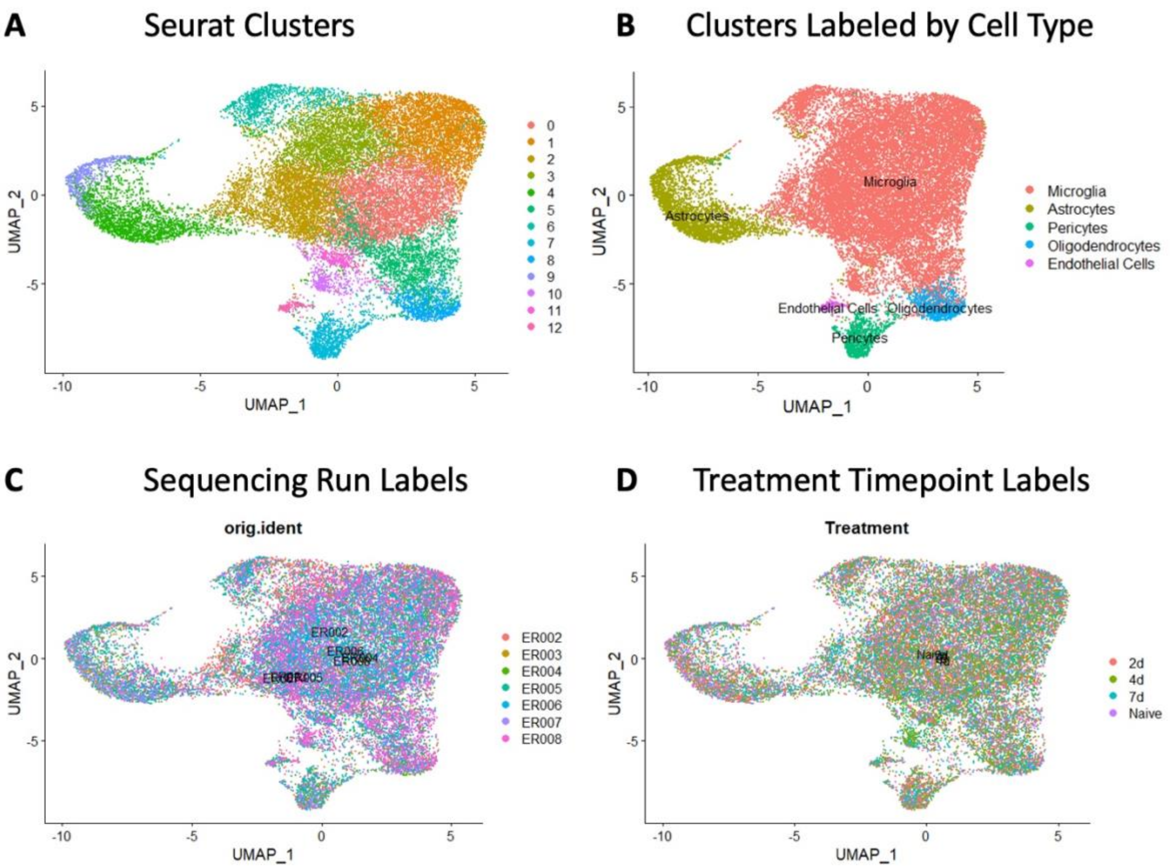

Figure S1: UMAP with all cells and labels showing unbiased distribution. UMAPs including all sequenced cells are shown with Seurat-identified clusters (A), cell-type labels as identified by canonical markers (B), the sequencing run from which the cells originated (C) and the treatment timepoint (D). Cells isolated from N=32 mice were used to generate this figure. Related to STAR Methods.

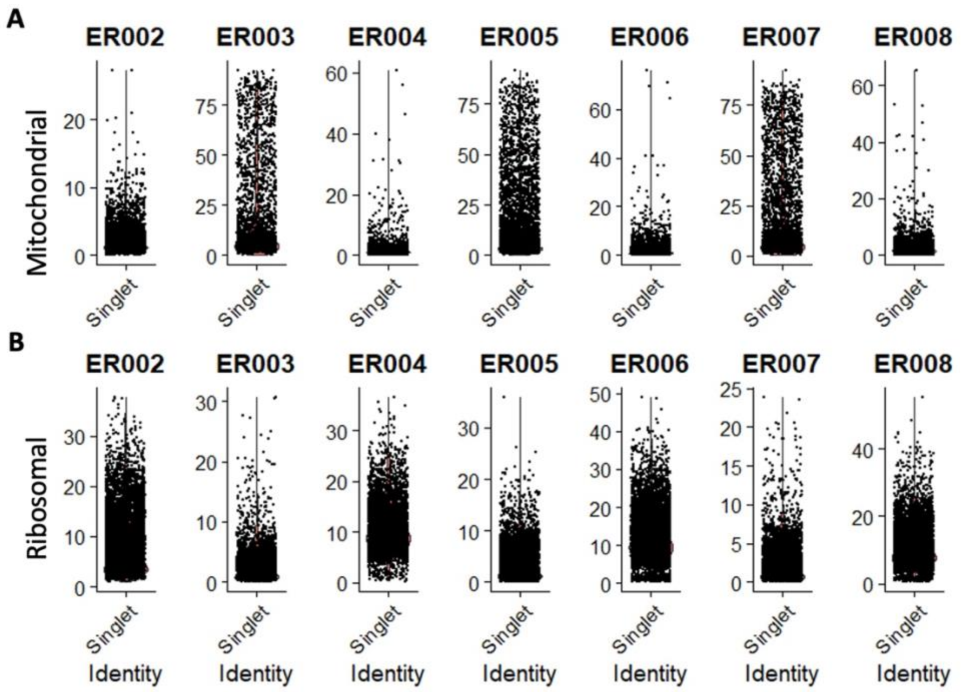

Figure S2: Pre-quality check sequencing run mitochondrial and ribosomal gene distribution. The distribution of mitochondrial (A) and ribosomal (B) genes from each sequencing run is shown before QC filtering. Cells isolated from N=32 mice were used to generate this figure. Related to STAR Methods.

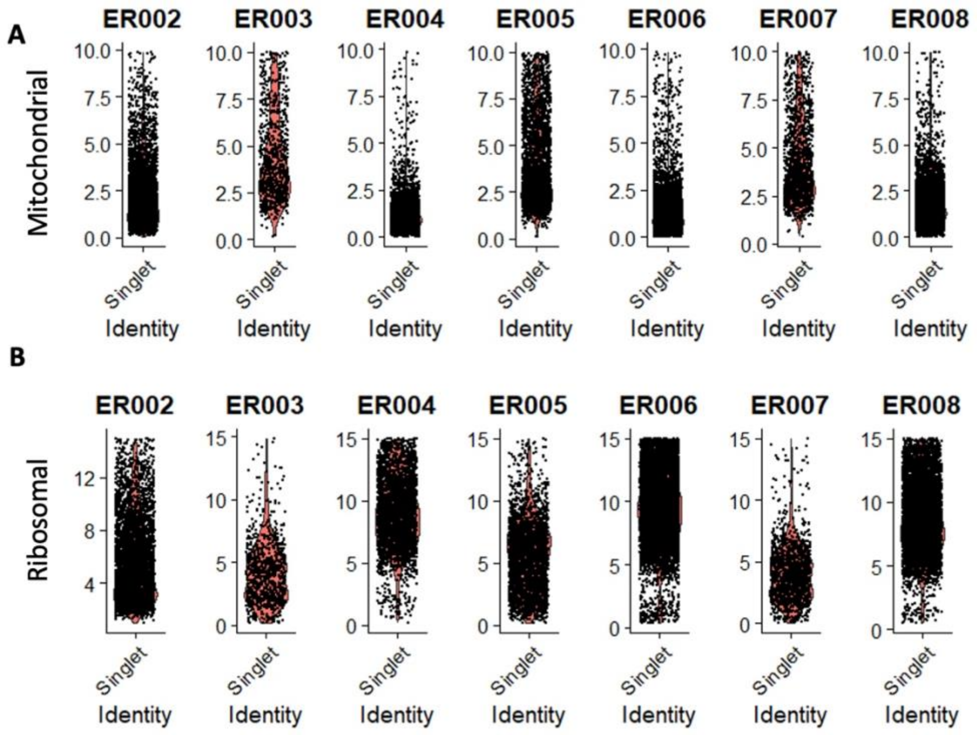

Figure S3: Post-quality check sequencing run mitochondrial and ribosomal gene distribution. The distribution of mitochondrial (A) and ribosomal (B) genes from each sequencing run is shown after QC filtering. Cells isolated from N=32 mice were used to generate this figure. Related to STAR Methods.

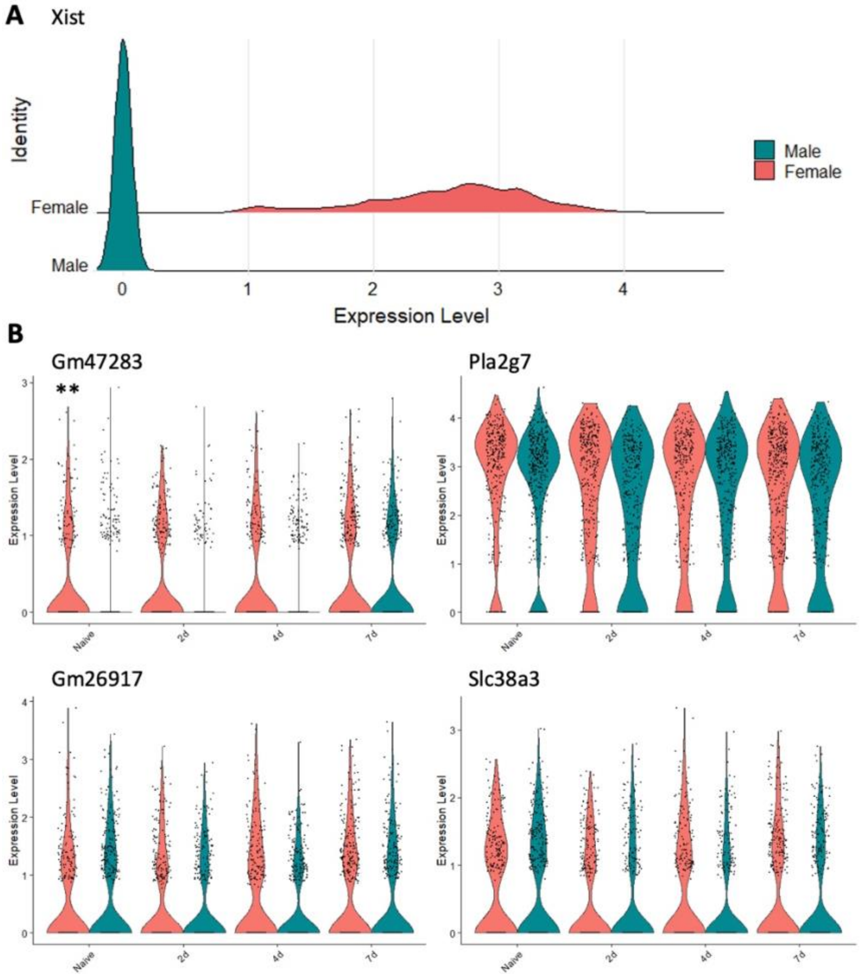

Figure S4: Male and female gene expression signatures do not differ significantly after FUS-BBBO. (A) Astrocytes isolated from male and female mice were identified based on their expression of *Xist*. (B) Four genes that exhibited differential gene expression between male and female astrocytes at various timepoints. Significant differences between male and female astrocytes within each treatment timepoint were evaluated by Wilcoxon rank sum test. ** P ≤ 0.01. Cells isolated from N=32 mice were used to generate this figure. Related to STAR Methods.

Supplementary Table 1. Sub-cluster Gene Ontology terms. Cells isolated from N=32 mice were analyzed to generate this table. Related to Figure 6.

Cluster	GO.ID	Term	Fisher P-Value
0	GO:0051239	regulation of multicellular organismal process	5.30E-14
	GO:0065008	regulation of biological quality	1.25E-12
	GO:0051049	regulation of transport	3.72E-10
	GO:2000026	regulation of multicellular organismal development	4.32E-10
	GO:0007399	nervous system development	5.14E-10
	GO:0050793	regulation of developmental process	1.10E-09
1	GO:0006820	anion transport	1.18E-04
	GO:0007155	cell adhesion	2.24E-04
	GO:0065008	regulation of biological quality	3.78E-04
	GO:0008284	positive regulation of cell population proliferation	1.03E-03
	GO:0007409	axonogenesis	2.84E-03
	GO:0048812	neuron projection morphogenesis	3.00E-03
2	GO:0006811	ion transport	7.06E-06
	GO:0006812	cation transport	1.43E-03
	GO:0034220	ion transmembrane transport	3.70E-03
	GO:0055085	transmembrane transport	4.16E-03
	GO:0030900	forebrain development	1.09E-02
	GO:0007399	nervous system development	2.91E-02
3	GO:0099177	regulation of trans-synaptic signaling	4.23E-05
	GO:0099536	synaptic signaling	1.02E-04
	GO:0034330	cell junction organization	1.62E-04
	GO:0099537	trans-synaptic signaling	1.98E-04
	GO:0051049	regulation of transport	2.17E-04
	GO:0007268	chemical synaptic transmission	4.37E-04
4	GO:0065008	regulation of biological quality	1.91E-05
	GO:0050896	response to stimulus	5.09E-04
	GO:0051716	cellular response to stimulus	2.00E-03
	GO:0048468	cell development	7.30E-03
	GO:0022008	neurogenesis	7.85E-03
	GO:0042063	gliogenesis	3.39E-02
5	GO:0006928	movement of cell or subcellular component	8.48E-07
	GO:0040011	locomotion	1.40E-06
	GO:0034329	cell junction assembly	6.37E-06
	GO:0048870	cell motility	7.57E-06
	GO:0048667	cell morphogenesis involved in neuron differentiation	1.43E-05
	GO:0061564	axon development	7.03E-05
